# Prevalence, aetiology and risk factors of mastitis of dairy cows kept under extensive management system in west Wollega, western Oromia, Ethiopia

**DOI:** 10.1002/vms3.503

**Published:** 2021-05-06

**Authors:** Gutu Kitila, Bedaso Kebede, Moti Wakgari

**Affiliations:** ^1^ Ethiopian Public Health Institute Addis Ababa Ethiopia; ^2^ Veterinary Drug and Animal Feed Administration and Control Authority Addis Ababa Ethiopia; ^3^ College of Health Sciences Addis Ababa University Addis Ababa Ethiopia; ^4^ Bedelle Regional Veterinary Laboratory Center Bedelle Ethiopia; ^5^ College of Agriculture and Veterinary Medicine Jimma University Jimma Ethiopia

**Keywords:** bovine, causative agents, CMT, mastitis, prevalence, udder

## Abstract

**Introduction:**

Mastitis is an inflammation of the mammary gland that could either be clinical or subclinical, and can be caused by infectious agents. It has different aetiological pathogens such as *Staphylococcus* *aureus*, *Streptococcus* *agalactiae*, *Escherichia coli* that pose different economic and health impacts. In Ethiopia, previous studies on mastitis have been focused on semi‐intensive and intensive dairy production system. The objectives of this study were therefore to estimate prevalence, aetiology of causative pathogens and identify different risk factors of mastitis in extensive management systems. A total of 532 lactating cows were randomly selected for a cross‐sectional study carried out in four districts of west Wollega, western Oromia, Ethiopia.

**Result:**

Based on the clinical examination and California mastitis test, 39.67% overall prevalence of mastitis on cow level was recorded. Of them, 16.70% and 22.70% were clinical and subclinical mastitis, respectively. Among 2,128 quarters examined 210 were blind teats. The associated risk factors age ≥8 years (16.35%), parity 1–3 calving (27.63%), milk yield 1–2 litres (21.98%), lactation stage <4 months (18.04%) and tick infestation (26.70%) were significantly associated in the occurrence of mastitis. *Staphylococcus* spp. (15.60%) were the dominant bacteria isolated from collected mastitic milk.

**Conclusion:**

Mastitis is an important disease for dairy cows kept under an extensive management system. Its occurrence is determined by risk factors like age, parity, milk yield, lactation stage and tick infestation. This study is indicated *Staphylococcus* species as the major bacteria isolated from mastitic milk. This study warrants further study on the prevalence, aetiology, economic impact and potential risk factors of mastitis in dairy cows kept in extensive production system.

## 1 INTRODUCTION

Dairy production plays a great role in the conversion of feed and roughages to nutritious milk (Yohannes, 2003). Urban and peri‐urban dairy farms are enclosed in semi intensive and intensive management systems because of high human population density, accessibility to technology input, high demand for animal products and purchasing power in the urban centre. But, extensive management systems of dairy farms focus on rural areas because of dairy cows kept in pastolarist area, as secondary income in cropping areas and poor genetic performances (Yoseph et al., 1998). According to FAO (2003), 42% of the total cattle herds for the private holdings are milking cows. However, milk production often does not satisfy the country's milk requirements due to a multitude of associated factors such as poor genetic performances, sub optimal feeding and diseases. Mastitis, known to be a complex and costly disease of dairy cows, results from the interaction of the cow and environment including milking processes and microorganisms (Azmi et al., 2008).

Mastitis is an inflammation of the mammary gland that could either be clinical (CM) or subclinical (SCM), infectious or non‐infectious (Markey et al., 2013). It creates an economic impact and zoonotic importance (Gonzalez & Wilson, 2003; Seegers et al., 2003). The occurrence of mastitis is determined due to the presence of contagious pathogens (*Staphylococcus* *aureus*, *Streptococcus* *agalactiae*, *Mycoplasma* spp. and *Corynebacterium bovis*) and environmental pathogens (*Escherichia coli*, *Klebsiella* spp., *Streptococcus* *dysgalactiae* and *Streptococcus* *uberis*) (Smith et al., 1985; Harmon, 1994; Radostitis et al., 2007; Cervinkova et al., 2013). The CM is characterized by the presence of the cardinal signs of an inflammation, while SCM does not show visible signs on the udder (Khan & Khan, 2006; FAO, 2014).

In Ethiopia, various studies reported on the prevalence, aetiology and risk factors of mastitis in dairy farms managed by semi‐intensive and intensive systems (Abera et al., 2012; Almaw et al., 2008; Seyoum et al., 2017; Tadesse & Chanie, 2012). However, no study was conducted on the dairy cows kept under extensive management system where the cows are often provided with natural pasture and agricultural by‐products and occasionally supplemented with imported feed. Therefore, the aim of the current study was to study the prevalence, aetiology of causative pathogens and risk factors of clinical and subclinical mastitis of lactating cows kept under the extensive system.

## 2 METHODS AND MATERIALS

### 2.1 Study animals and design

The cross‐sectional study was conducted from January to June 2017 in local zebu dairy cows in selected four districts such as Lalo Asabi, Guliso, Nejo and Manasibu of west Wollega zone, Ethiopia.

### 2.2 Sample size and sampling method

The cows selected were those managed under an extensive production system. From four districts 110 herds were selected and 4–5 cows per herd were involved in the study. A simple random sampling method was used for sampling representative study animals. The sample size was determined according to Thrusfield (2005) at a 95% confidence interval and 5% precision with the expected mastitis prevalence of 50% because of no study conducted before in the study area. Therefore, after substituting the above‐mentioned values, 384 was the sample size. However, to increase the precision values the sample size was increased to 532.

### 2.3 Detection of mastitis

Mastitis was detected using the California mastitis test (CMT) and clinical inspection of the udder (Quinn et al., 2004). The clinical inspections are conducted through visual examination of udder and changes in milk. Moreover, udder palpation to detect fibrosis, cardinal signs of inflammation, tick infestation, and swelling of the supra mammary lymph nodes. Subclinical mastitis diagnosis was based on CMT results where nature of coagulation and viscosity of the mixture (milk and CMT reagent) indicates the presence and severity of the inflammation (Harmon, 1994). From each quarter of the udder, a squirt of milk sample was placed in each of the cups on the CMT paddle and an equal amount of 3% CMT reagent was added to each cup and mixed well. Reactions were graded as 0 and trace for negative, +1, +2 and +3 for positive to subclinical mastitis (NMC, 1990 and Quinn et al., 2004).

### 2.4 Sample collection, handling and storages

Milk samples were collected by a standard milk‐sampling technique from all teats with CM and SCM (NMC, 1990). The udder, especially the teats were cleaned and dried before milk sample collection. Dust, particles of bedding and other filth were removed by brushing the surface of the teats and udder with a dry towel. The teats were washed with tap water and dried. Then the teats were swabbed with cotton, soaked in 70% alcohol (NMC, 1990). To prevent recontamination of teats during scrubbing with alcohol, teats on the far side of the udder were scrubbed with alcohol first, then those on the near side. Then to reduce contamination of the teat ends during sample collection, the teats were sampled first followed by the far ones. Ten millilitres of milk samples were collected into a sterile test tube after discarding the first three milking squirts. Then samples were placed in racks for ease of handling and transported in an icebox to the microbiology laboratory of Bedelle regional veterinary laboratory centre and stored at 4°C for a maximum of 24 hr until inoculated on a standard bacteriological media (Biru, 1989; NMC, 1990).

### 2.5 Bacteriological isolation and characterization

Milk samples were bacteriologically examined according to the procedures employed by Quinn et al., (2004). In refrigerated milk samples, bacteria may be concentrated in the creamy layer and held within clumps of fat globules (NMC, 1990). Hence, the dispersion of fat and bacteria was accomplished by warming the samples at 25°C for 15 min and shaking before plating on a standard bacteriological media. A loopful of milk sample collected from each quarter was inoculated separately on to MacConkey agar and blood agar base enriched with 7% defibrinated bovine blood. The inoculated plates were then incubated aerobically at 37°C for 24 hr. When growth was not observed after incubation for 24 hr, the quarter's milk sample was reinoculated on an enriched tryptone‐soya broth to amplify the bacterial growth. Identification of the bacteria on primary culture was made on the basis of colony morphology, haemolytic characteristics, Gram stain reaction including shape and arrangements of the bacteria, catalase and O‐*F* tests. Gram‐negative isolates grown on MacConkey agar were identified based on growth characteristics on MacConkey agar, oxidase reaction, catalase test, tripleugar iron agar (Quinn et al., 2004).

### 2.6 Data collection and analysis

All the collected data including age, parity, breed, lactation stage, tick infestation and milk yield were recorded. Depending on clinical inspection and CMT results, cases were categorized as either positive or negative; positive cases were further categorized as clinical and subclinical mastitis. The age of the study animals was determined from dental examination and categorized 3 to 5 years, 6 to 7 years and 8 and above years. Parity was categorized as 1–3 calves, 4–6 calves and calves 7 and above. Milk yield was categorized as <1 litre, 1–2 litres, 3–5 litres, and ≥6 litres. The lactation stage was categorized as <4 months, 4–8 months and ≥8 months. Tick infestation could be either presence or absence. The data were recorded in the Microsoft Excel spreadsheet and SPSS version 20 used in statistical analysis. The descriptive statistics and chi‐square tests were used to determine the prevalence and association of risk factors and aetiology with the occurrence of bovine mastitis. The association is statistically significant if *p*‐value <.05.

## 3 RESULTS

The overall prevalence of mastitis at the cow level was 39.67% (210/532) of which CM and SCM were 16.70% and, 22.70%, respectively. The prevalence of CM and SCM were increasing as the age of cows increased. The prevalence of CM and SCM was higher in parity of 1–3 calving than in others. Moreover, the prevalence of mastitis was higher in cows with 1–2 litres of milk yield than the rest. The prevalence of CM was higher in lactation stage of less than four months, but SCM was higher in lactation stage between four and eight months than that of the rest. Tick infestation presence in both CM and SCM was associated with mastitis compared to absence of infestation. All risk factors were significantly associated (*p* < .05) with the occurrence of mastitis (Table 1).

**TABLE 1:** Associated risk factors for the occurrence of clinical and subclinical mastitis

Risk factors	Mastitis				X^2^‐value	*p*‐value
	Clinical	Subclinical	Total infected	Total prevalence (%)		
Age (years)					8.343^a^	.015
3–5	19 (3.60%)	25 (4.70%)	44	8.27		
6–7	34 (6.40%)	45 (8.50%)	79	14.85		
≥8	36 (6.80%)	51 (9.60%)	87	16.35		
Total	89 (16.70%)	121 (22.70%)	210	39.47		
Parity					9.264^a^	.010
1–3 calving	63 (11.80%)	84 (15.80%)	147	27.63		
4–6 calving	25 (4.70%)	36 (6.80%)	61	11.46		
≥7 calving	1 (0.20%)	1 (0.20%)	2	0.376		
Total	89 (16.70%)	121 (22.70%)	210	39.47		
Milk yield					9.559^a^	.023
less 1 litre	35 (6.60%)	50 (9.40%)	85	15.97		
1–2 litres	50 (9.40%)	67 (12.60%)	117	21.98		
3–5 litres	4 (0.80%)	2 (0.40%)	6	1.12		
≥6 litres	0 (0.00%)	2 (0.40%)	2	0.4		
Total	89 (16.70%)	121 (22.70%)	210	39.47		
Lactation stage					9.628^a^	.008
Less than 4 months	56 (10.50%)	40 (7.50%)	96	18.04		
4–8 months	22 (4.10%)	49 (9.20%)	71	13.35		
≥8 months	11 (2.10%)	32 (6.00%)	43	8.08		
Total	89 (16.70%)	121 (22.70%)	210	39.47		
Tick infestation					8.555^a^	.003
Absent	21 (3.90%)	47 (8.80%)	68	12.77		
Present	68 (12.80%)	74 (13.90%)	142	26.70		
Total	89 (16.70%)	121 (22.70%)	210	39.47		

Note

^a^ significantly associated.

The overall prevalence of mastitis at the quarter level was 20.35% of which 9.87% was blocked teats. The prevalence of the positive CMT was higher on the left front of the quarter of udder than others (Table 2). Bacteria isolated from mastitic milk samples were *Staphylococcus* species (15.60%), *Enterobacteriaceae* (8.80%), *Micrococcus* species (3.60%) and *Rhodococcus equi* (0.80%). Bacteria isolated were significantly associated with the occurrence of mastitis (Table 3). The district level prevalence of mastitis was higher in Nejo district (12%) than in others (Table 4).

**TABLE 2:** Prevalence of mastitis at the quarter's level in dairy cows

Observational level	Number examined	CMT result		Blind teats
		+ve	‐ve	
Quarters				
Right hind	532	53 (10.00%)	403 (75.80%)	76 (14.30%)
Right front	532	53 (10.00%)	433 (81.40%)	46 (8.60%)
Left hind	532	53 (10.00%)	429 (80.60%)	50 (9.40%)
Left front	532	64 (12.00%)	430 (80.80%)	38 (7.10%)
Total quarter	2,128	223 (10.48%)	1695 (79.65%)	210 (9.87%)

**TABLE 3:** Isolation of bacteria from clinical and subclinical mastitis

Bacterial	Clinical	Subclinical	Total prevalence	X^2^‐value	*p*‐value
*Staphylococcus* spp.	19 (3.60%)	64 (12.00%)	83 (15.60% )	13.315^a^	.010
Enterobacteriaceae	9 (1.70%)	38 (7.10%)	47 (8.80%)		
*Micrococcus* spp.	4 (0.80%)	15 (2.80%)	19 (3.60%)		
*Rhodococcus equi*	0 (0.00%)	4 (0.80%)	4 (0.80%)		
Total	32 (6.10%)	121 (22.70%)	153 (28.80%)		

Note

N.B. Spp. = Species.

**TABLE 4:** Prevalence of clinical and subclinical mastitis at the districts level

Districts	Clinical	Subclinical	Total prevalence
Lalo Asabi	21 (3.90%)	27 (5.10%)	48 (9.00%)
Guliso	21 (3.90%)	26 (4.90%)	47 (8.80%)
Nejo	30 (5.60%)	34 (6.40%)	64 (12%)
Manasibu	17 (3.20%)	34 (6.40%)	51 (9.60%)
Total	89 (16.73%)	121 (22.74%)	210 (39.47%)

## 4 DISCUSSION

The current study revealed that the overall prevalence of mastitis at the cow level was 39.67%. It is similar with the findings from in and around Addis Ababa of urban and peri‐urban dairy production system (Babaei et al., 2007), North Gondar dairy farm (Nibret, 2007), dairy farms in central highland of Ethiopia (Hunderra et al., 2005) and Addis Ababa dairy farms (Tadesse and Chanie). In the present study, mastitis prevalence was lower than previous study reported from Sebeta, central Ethiopia (Sori et al., 2005), Hawassa, southern Ethiopia (Abebe et al., 2016), Asella, southern Ethiopia (Lakew et al., 2009) and Holeta, central Ethiopia (Mekibeb et al., 2010). It is because of difference in the breeds (local), county, production system (extensive), housed, floor type (field for extensive system) and milk yield (Almaw et al., 2008). In the current study, CM and SCM prevalence was 16.70% and 22.70%, respectively. The differences might be contributed from the fact that clinical mastitis can easily be diagnosed and treated (Motaung et al., 2017), while subclinical form has no visible abnormalities which is hardly diagnosed by the farmers and progresses to be a source of infection in the extensive farm (Abebe et al., 2016; Ismael, 2018). Moreover, ineffective mastitis control programmes and poor hygiene standards in the study areas might have been a key contributor (Abrahmsén et al., 2014). Of the 2,128 quarters examined, 210 were blind teats and the prevalence of mastitis at the quarter level was higher in left front quarters. Even if there is no immediate explanation established for this observation, it is probably in the process of milking left front quarters were milked first before the other quarters because most of the operators tend to be right handed and sit first to the left of the animals (Shittu et al., 2012). This finding agree with the report from Nigeria (Shittu et al., 2012), but contrary to the study of Abebe et al., (2016) reported more bovine mastitis in the hind quarters.

Bacteria isolated from collected mastitic milk were *Staphylococcus* species (15.60%), Enterobacteriaceae (8.80%), *Micrococcus* species (3.60%) and *Rhodococcus equi* (0.80%) and it was significantly associated with the occurrence of mastitis. Previous study findings by Almaw et al., (2008), Abera et al., (2012) and Lakew et al., (2009) reported isolation of these pathogens from mastitic milk samples. The current study report on higher prevalence of *Staphylococcus* species is in agreement with the findings by Hunderra et al., (2005), Mekibeb et al., (2010), Harini and Sumathi (2011) and Tadesse and Chanie (2012), but they were different in management system from the current study which is extensive management system.

The dominance of *Staphylococcus* species in this study is probably because it is attributed to the wide distribution inside mammary glands and on the skin of teats and udders and transmitted between dairy cows during hand milking (Jones et al., 1998). *Staphylococcus* species have adapted to survive in the udder and establish chronic and subclinical inflammations. It sheds into the milk to serve as a source of infection for healthy cows during the milking process (Radostitis et al., 2007). The high prevalence of this organism may be due to the frequent colonization of the teat, its ability to exit intracellularly and localize within micro‐abscesses in the udder and its resistance to antibiotic treatment (Belay et al., 2012).

In this study, increased age was associated with the occurrence of mastitis which agrees with the findings of Dego and Tareke (2003) and Abera et al., (2013) who found that the risk of mastitis increases significantly with the advancing age of the cows. Clinical mastitis prevalence was higher in the early lactation stage less than four months which agree with the reports of Mungube et al., (2004) and Biffa et al., (2005). However, SCM prevalence was higher in the medium lactation stage between 4 to 8 months and it agrees with the reports of Nesru (1999), Kerro & Tareke, 2003 and Alemu et al., 2013. Augmentation of mastitis prevalence with increasing age and lactation stage can be due to increasing ease of penetration of the teat duct by pathogens and previously accumulated pathogens (Radostitis et al., 2007). In the current study the prevalence of mastitis was higher for the parity factor in cows with 1–3 calving (lower parity) than other groups because small number of cows were involved in the other groups of parity factor. This finding agrees to the reports of Mungube et al., (2004), Biffa et al., (2005), Getahun et al., (2008), Lakew et al., (2009) and Alemu et al., (2013). The presence of tick infestation was associated with mastitis, hence Alemu et al., (2013) found that the presence of tick infestation increased prevalence of mastitis, but it was insignificantly associated. Tick infestation is probably a predisposing factor for the occurrence of mastitis. Medium milk yields which are 1–2 litres was associated with mastitis compared to higher milk yields and contrary to this, Radostitis et al. (2007) entail that high‐yielding cows are associated with the occurrence of mastitis. The difference could be because of small number of cows with higher milk yields were involved in the current study. The highly productive cows’ mammary gland teats canal could slack and easily enter pathogens into the udder, and finally produce infection (Muluye et al., 2017).

## 5 CONCLUSION

The study indicated that mastitis is an important disease for dairy cows kept under an extensive management system. SM was higher in prevalence than CM. *Staphylococcus* species were the most dominant bacteria isolated. Age, milk, yield, lactation stage, parity and tick infestation were important risk factors associated with the occurrence of mastitis. Therefore, this study warrants the creation of awareness on the improvement of hygiene and prevention of injury on udder and teats and further study on risk factors, aetiology and prevalence of mastitis of dairy kept under an extensive management system.

## CONFLICT OF INTERESTS

The authors declare that they have no conflicts of interests.

## AUTHOR CONTRIBUTION

**Gutu Kitila:** Conceptualization; Investigation; Methodology; Project administration; Resources; Supervision; Writing‐review & editing. **Bedaso Kebede Kebede:** Conceptualization; Formal analysis; Investigation; Software; Writing‐original draft; Writing‐review & editing. **Moti Wakgari:** Conceptualization; Investigation; Methodology; Project administration; Resources; Supervision; Writing‐review & editing.

## ETHICS APPROVAL AND CONSENT TO PARTICIPATE

A local ethics committee ruled that no formal ethics approval was required to conduct this research. Before conducting the research, informed consent was obtained from the owners of the lactating cows used in this study.

### PEER REVIEW

The peer review history for this article is available at https://publons.com/publon/10.1002/vms3.503.

## Data Availability

All data generated and analysed during this study are included in this published article.
